# SIRT2 suppresses expression of inflammatory factors via Hsp90‐glucocorticoid receptor signalling

**DOI:** 10.1111/jcmm.15365

**Published:** 2020-06-09

**Authors:** Kai Sun, Xuan Wang, Na Fang, Ao Xu, Yao lin, Xiaofang Zhao, Adil J. Nazarali, Shaoping Ji

**Affiliations:** ^1^ Department of Hematology Henan Provincial People's Hospital Henan University Henan China; ^2^ Department of Biochemistry and Molecular Biology School of Basic Medical Sciences Henan University Henan China; ^3^ Jiangsu Superbio Co., Ltd Nanjing China; ^4^ College of Pharmacy and Nutrition and Neuroscience Research Cluster University of Saskatchewan Saskatoon SK Canada

**Keywords:** deacetylation, Glucocorticoid receptor, Hsp90, inflammatory cytokine, SIRT2

## Abstract

SIRT2 is a NAD^+^‐dependent deacetylase that deacetylates a diverse array of protein substrates and is involved in many cellular processes, including regulation of inflammation. However, its precise role in the inflammatory process has not completely been elucidated. Here, we identify heat‐shock protein 90α (Hsp90α) as novel substrate of SIRT2. Functional investigation suggests that Hsp90 is deacetylated by SIRT2, such that overexpression and knock‐down of SIRT2 altered the acetylation level of Hsp90. This subsequently resulted in disassociation of Hsp90 with glucocorticoid receptor (GR), and translocation of GR to the nucleus. This observation was further confirmed by glucocorticoid response element (GRE)‐driven reporter assay. Nuclear translocation of GR induced by SIRT2 overexpression repressed the expression of inflammatory cytokines, which were even more prominent under lipopolysaccharide (LPS) stimulation. Conversely, SIRT2 knock‐down resulted in the up‐regulation of cytokine expression. Mutation analysis indicated that deacetylation of Hsp90 at K294 is critical for SIRT2‐mediated regulation of cytokine expression. These data suggest that SIRT2 reduces the extent of LPS‐induced inflammation by suppressing the expression of inflammatory factors via SIRT2‐Hsp90‐GR axis.

## 1 INTRODUCTION

SIRT2 is an NAD^+^‐dependent deacetylase that belongs to the Sirtuin family, which consists of 7 members in humans(SIRT1‐7). Recent studies have revealed that SIRT2 is involved in multiple cell processes, including energy metabolism, cell proliferation, cell differentiation, apoptosis, inflammation, lifespan and tumorigenesis.^1^ Over the past few years, a number of SIRT2 substrates have been identified, ranging from histones to non‐histone proteins.

Most notably, SIRT2 has been implicated in the maintenance of genomic integrity^2^, ^3^, ^4^ and in the regulation of the cell cycle and cell differentiation.^5^, ^6^, ^7^, ^8^, ^9^, ^10^ We have previously demonstrated that SIRT2 promoted differentiation of an oligodendroglia CG4 cell line.^11^ SIRT2 appears to also play an important role in energy metabolism. For example, SIRT2 deacetylates and activates PGAM (glycolytic enzyme phosphoglycerate mutase), which results in a decrease in cellular NADPH levels.^12^, ^13^ Moreover, SIRT2 can deacetylate G6PD (glucose‐6‐phosphate dehydrogenase), in which deacetylation of K403 results in the formation of an active dimer, leading to production of cytosolic NADPH.^14^ SIRT2 has also been shown to deacetylate HIF‐1α under hypoxic conditions, leading to HIF‐1α hydroxylation and ubiquitination followed by degradation.^15^

More recently, there has been increasing evidence that indicates SIRT2 is involved in inflammatory processes. In animal models of collagen‐induced arthritis, SIRT2 inhibits the inflammatory response and relieves physical impairment.^16^ Remarkably, expression level of SIRT2 mRNA was dramatically decreased in extracellular plasma of rheumatoid arthritis patients compared with the healthy controls.^17^ Moreover, a cell permeative fusion protein, PEP‐1‐SIRT2, significantly blocks the expression of cytokines under lipopolysaccharide (LPS) stimulation.^18^ However, some results are controversial. For example, repression of SIRT2 activity has been shown to down‐regulate LPS‐induced inflammatory response via TNF‐α and IL‐6,^19^ and knock‐down of SIRT2 has also been shown to repress microglial activation induced by LPS.^20^ Overall, there is a tendency for data to support a role for SIRT2 in the repression of an excessive immune activation.

SIRT2 has been shown to deacetylate histone H3K18 in the immune response against bacterial infection^21^ and, in turn, modulate the expression of anti‐inflammatory genes. Other recent reports demonstrate that SIRT2 can repress the inflammatory response via deacetylation and inhibition of NF‐kB, using transgenic (SIRT2^‐/‐^) mice, in models of sepsis or colitis.^22^, ^23^, ^24^ Repression of SIRT2 expression via forced expression of miR‐339 increases the acetylation level of NF‐kB, which may participate in the stress‐induced immune response.^25^ However, other than H3 and NF‐kB, SIRT2 exhibits the ability to antagonize inflammation by previously undetermined mechanisms.

In the present study, we identified Hsp90 as a novel substrate of SIRT2. Hsp90 is known to regulate GR translocation to nucleus, modulating the expression of downstream genes. Under resting conditions, Hsp90 and GR form a complex within the cytoplasm. Typically, glucocorticoid stimulation leads to the dissociation of the GR from Hsp90. This allows GR to form homodimers and translocate to the nucleus, where it forms heterocomplex with NF‐kB, preventing the transcriptional activation of inflammatory genes.^26^ Inhibition of Hsp90 also attenuates activation of NF‐kB and inhibits expression of its downstream genes,^27^, ^28^ indicating a regulatory role for Hsp90 in the NF‐kB‐dependent inflammatory response. Accordingly, secreted Hsp90 alpha (eHsp90α) into the extracellular stromal matrix promotes an inflammatory reaction in the tumour microenvironment in an MEK‐ERK‐ and NF‐kB‐dependent manner.^29^ Acetylated rat Hsp90α at K294 (Scroggins BT et al, but site corresponds to K295 in new version of rat Hsp90α: NP_786937) promotes its binding to its interacting partners^30^; therefore, we speculated that SIRT2 may repress immune reaction via Hsp90‐GR signalling. Our results provide new insights into the regulation of the inflammation via SIRT2.

## 2 MATERIALS AND METHODS

### 2.1 Hsp90 mutation and plasmid construction

The coding sequence of rat SIRT2 (rSIRT2) was amplified from a previously constructed SIRT2‐pIRES2‐EGFP vector^11^ with the upstream and downstream primers 5′‐GGATCCATGGACTTCCTACGGAATTT‐3′ and 5′‐CTCGAGCTAGTG TTCCTCTTTCTCTT‐3′, respectively. The product was cloned into T‐easy vector (Promega) for verification of sequencing, and then, the coding region was subcloned into pGEX‐6P‐1 vector via restriction sites EcoR I and Xho I. Then, GST‐SIRT2 fusion protein was expressed in *E coli* using the SIRT2‐pGEX vector upon IPTG induction.

An overlap PCR was performed to yield an acetylation‐mimic mutation and acetylation‐null mutation of rat Hsp90α (rHsp90α) at K294. In brief, the upper and lower segments of rHsp90α were amplified by PCR from cDNA clone (OriGene, Rockville, MD). Both segments are purified from an acrylamide gel and mixed together with Phusion DNA polymerase (Bio‐Rad, CA), PCR buffer and dNTP. The resultant PCR reaction was amplified for 25 cycles, and then, extreme upstream and downstream primers were added into the PCR reaction for an additional 25 cycles. rHsp90 primers for the acetylated‐mimic mutation (K294Q) overlap PCR: upper fragment (reverse) 5′‐TCCAGATCGG**CTG**TGTTTTGTTGAG‐3′ and lower fragment (forward) 5′‐CTCAACAAAACA**CAG**CCGATCTGGA‐3′. rHsp90 primers for the acetylation‐null mutation (K294R) overlap PCR: upper fragment (reverse): 5′‐TCCAGATCGG**CCT**TGTTTTGTTGAG‐3′ and lower fragment (forward): 5′‐CTCAACAAAACA**AGG**CCGATCTGGA‐3′. The extreme upstream and downstream primers for both K294Q and K294R mutations were 5′‐GCTAGCATGCCTGAGGAAACCCAGACCC‐3′ and 5′‐CTCGAGTCATC AATACCTAGACCAAGC‐3′. The full‐length Hsp90α mutants were cloned into T‐easy vector to verify by DNA sequencing. The verified Hsp90 mutants were subcloned into pcDNA3.1/Myc‐His A with NheI and XhoI enzyme sites.

### 2.2 Protein expression and purification

SIRT2‐pGEX and pGEX‐6P‐1 plasmids were transformed into BL21 competent cells. A single colony was picked up and cultured in LB medium overnight. The cultured cells were inoculated to fresh LB at 1:200 with ampicillin and cultured for 2‐4 hours. These cultures were then cooled to room temperature for 30 minutes, and 1.0 mmol/L of IPTG was added. The induced cells were cultured at 26℃ for 4 hours and then collected by centrifugation at room temperature. The cell pellet was lysed in PBS (0.8% NaCl, 0.02% KCl, 0.144% Na_2_HPO_4_, 0.024% KH_2_PO_4_, 1.0 μg/mL phenylmethylsulphonyl fluoride, pH 7.4) by one freeze/thaw cycle at − 80℃ and subjected to sonication at 40% of maximum power for 3 × 20 seconds. Solubility of the GST‐fusion protein or GST was analysed by SDS‐PAGE and Coomassie staining. The soluble fusion protein in supernatant of the lysate was purified as described previously.^11^, ^31^ Briefly, glutathione Sepharose 4B beads (Thermo Fisher) were washed with PBS and added to the cleared lysate, incubated at 4°C for 1.0 hour and washed with PBS for 3 × 10 minutes by repeated centrifugation. Pellets were subjected to analysis by SDS‐PAGE and Coomassie staining. The purified fusion protein was kept at 4°C for the following experiments.

### 2.3 RNA knock‐down and construction of lentiviral vectors

Three short RNAs against rSIRT2 were designed through BLOCK‐iT RNAi Designer (Invitrogen, San Diego, CA), including siRNA‐1:5′‐GGACUUCCUACGGAAUUUA‐3′, siRNA‐2:5′‐GCUACACGCAGAAUAUUGA‐3′ and siRNA‐3:5′‐GACUCCAAGAAGGCUUACA‐3′. The siRNAs were commercially synthesized (Qiagen, Valencia, CA), along with random RNA controls. To test the efficiency of SIRT2 knock‐down, siRNA1‐3 were transfected into B104 cells (Figure 3A). After confirmation of knock‐down efficiency, the corresponding sequence of siRNA‐3 against human SIRT2 (hSIRT2) mRNA, 5′‐GACUCCAAGAAGGCCUACA‐3′ which was termed siRNA‐III, was transfected into Jurkat cells to confirm its efficiency. siRNA‐3, siRNA‐III and SIRT2 cDNA were sent to GenePhama^32^ to construct dual promotor lentiviral vectors of SIRT2 knock‐down (pGLV3/H1/GFP + Puro vector) or SIRT2 overexpression (LV5 vector) that separately express GFP for tracking the expression status in cells. Ready‐to‐use viral particles were provided and stored at − 80°C.

### 2.4 Cell culture and transfection

B104, B103, U87(MG), SH‐SY5Y and Jurkat cell lines were purchased from ATCC (Manassas, VA). EC9706 is from Chinese Academy of Sciences Cell Bank. Cells are cultured in Dulbecco's modified Eagle's medium with 10% foetal bovine serum, penicillin and streptomycin at 37℃ under 5% CO_2_ and then detached by 0.25% trypsin/EDTA to pass directly into flasks or dishes for experiments. In some experiments, cells were treated with 10 ng/mL of LPS for 12 hours prior to harvesting or treated with 10 μmol/L of dexamethasone. For Hsp90 acetylation‐mimic or acetylation‐null assays or siRNA experiments, the plasmids or double‐stranded RNA was diluted into Lipofectamine‐2000 according to manufacturer's instructions and transfected into cells without antibiotics. The culture medium was replaced with fresh growth medium 12 hours after transfection. For lentiviral infection, SIRT2 overexpression or knock‐down viral particles were thawed on ice and then the appropriate amount was dropped into cells following manufacturer's instruction depending on the titre of viral particles. Infected cells were selected by puromycin for 2 weeks. Cultures were imaged using an Eclipse Ti‐E microscope (Nikon, Melville, NY) at 20 × magnification to determine infection rates and were either used immediately for experiments or frozen in liquid nitrogen.

### 2.5 Pull‐down assay and mass spectrometry

The purified fusion protein (see ‘Protein expression and purification’) attached to the glutathione beads was used to pull‐down SIRT2 interacting protein(s). In brief, cultured B104 cells were lysed in RIPA buffer (50 mmol/L Tris–HCl, pH7.4, 150 mmol/L NaCl, 1% NP‐40, 0.25% Na deoxycholate, 1.0 mmol/L EDTA and 1:200 protease inhibitor cocktail (Sigma, St. Louis, MO). After centrifugation and pre‐cleaning with glutathione beads, purified GST‐SIRT2 protein or purified GST was added to the supernatant and incubated at 4°C overnight with gentle wave shaking. The samples were washed with RIPA buffer 4 times by repeated centrifugation. Final pellets were subjected to SDS‐PAGE followed by Coomassie staining, and novel band(s) in the GST‐SIRT2 lane were excised and identified by mass spectrometry (National Research Council Plant Biotechnology Institute, Saskatoon, SK). Protein identification from spectra was performed with Mascot (Matrix Science).

### 2.6 Pull‐down and Co‐Immunoprecipitation (Co‐IP) with western blot

The GST‐SIRT2 fusion protein was purified as described above. B104 cell was treated with or without 10 ng/mL LPS for 12 hours prior to harvesting. Cultured B104 cells were lysed in RIPA buffer on ice for 10 minutes and vortexed intermittently. The sample was centrifuged at 12 000 g for 10 minutes at 4°C, and the supernatant was incubated with purified GST‐SIRT2 or purified GST overnight at 4°C. Subsequently, samples were washed in RIPA buffer without protease inhibitor 4 times by repeated centrifugation. The final pellets, along with the control cell lysate, were subjected to SDS‐PAGE. Hsp90 was detected with a specific anti‐Hsp90 antibody as described under ‘Western blot’. To immunoprecipitate the proteins of interest, the lysate of LPS treated or control cells was prepared as described above. In brief, the lysate was pre‐cleaned with protein‐A beads by incubating for 1.0 hour at 4°C followed by centrifugation. The protein concentration of the supernatant was adjusted to 1.5 μg/μL with RIPA buffer and incubated with 20 μg of the specific antibody or normal control IgG overnight at 4°C. The samples were washed in RIPA buffer 4 times by repeated centrifugation. The precipitated complex was subjected to analysis of protein interaction as described under ‘Western blot’.

### 2.7 Isolation of nuclei from cytoplasm

Nuclei were isolated from B104 cells with overexpression or knocked‐down of SIRT2, as described previously.^33^ B104 cells treated with or without LPS were harvested by trypsin‐EDTA and pelleted by centrifugation at 500 g for 3 min. Briefly, according to the instruction of NE‐PER Nuclear and Cytoplasmic Extraction Reagents (Thermo, MA), cells were lysed in CER I and then CERII was added to the samples. The supernatant (cytoplasm) and pellet (nuclei) were separated by centrifuge. Proteins in both fractions were subjected to SDS‐PAGE, and purity was assessed by antibodies against lamin B1 (nuclei) or β‐actin (cytoplasm) as described under ‘Western blot’.

### 2.8 GRE‐driven dual‐luciferase reporter assay

The binding ability of GR to glucocorticoid response element (GRE) was examined using a luciferase reporter gene assay as previously described.^34^ The reporter plasmids, negative control plasmids and positive control plasmids were transfected into B104 cells according to manufacturer instructions of Cignal CRE Reporter Assay Kit (Qiagen). In addition, the reporter plasmids were transfected into cells infected with lentiviral particles for SIRT2 overexpression or SIRT2 knocked‐down. Cells were counted and transfected in 24‐well plates in triplicate for each sample set. No additional glucocorticoid, such as dexamethasone, was added to the culture medium, as glucocorticoid within the serum serves as ligands. The luciferase activity was examined 48 hours after transfection with Dual‐Luciferase Reporter Assay System (Promega, Madison, WI) according to the manufacturer's protocol. Briefly, the sample cells were washed and harvested with trypsin and lysed in lysis buffer (0. 5% NP 40, 10 mmol/L Hepes, pH 7. 9, 1. 5 mmol/L MgCl_2_, 10 mmol/L KCl, 1:200 protease inhibitor cocktail). The cell supernatant was collected after centrifugation for measurement of luciferase activity in a 96‐well plate. The value of luciferase activity was collected and normalized by the ratio of firefly luciferase to renilla luciferase.

### 2.9 Real‐time quantitative PCR

Total RNA was isolated with TRIzol Reagent (Thermos Fisher, MA) from Jurkat cells with Sirt2 overexpression, Sirt2 knocked‐down or control cells. Cells were treated with or without LPS prior to assays. cDNA was synthesized using High‐Capacity cDNA Reverse Transcription Kit (Thermos Fisher) according to the manufacturer's protocol. The mature mRNA levels of human *TNFα*, *IL‐1β* and *IL‐6* were detected by quantitative PCR using StepOnePlus RT‐PCR instrument (Applied Biosystems, Foster City, CA). The primers used were as follows: human *TNFα* forward primer 5′‐CTGAGGCCAAGCCCTGGTATG‐3′ and reverse primer 5′‐CTCCTCACAGGGCAATGATCC‐3′; human *IL‐1β* forward primer 5′‐GATAAGCCCACTCTACAGCTG‐3′ and reverse primer 5′‐AGGAAGACACAAATTGCATGG‐3′; human *IL‐6* forward primer 5′‐ATGCTACATTTGCCGAAGAGC‐3′ and reverse primer 5′‐CAGAGCTGTGCAGATGAGTAC‐3′; *GAPDH* forward primer 5′‐AGCAAGGCACCACTAGCCACC‐3′ and reverse primer 5′‐TGTTCCTCTTTCTCTTTGGTC‐3′. The relative expression levels were determined by threshold cycle differences (2^−ΔΔCT^) normalized to *GAPDH*.

### 2.10 Western blot

Cultured cells were harvested by trypsin‐EDTA, pelleted and resuspended in the RIPA buffer on ice for 10 minutes, or RIPA buffer was directly added into the flask, and cells were collected by cell scraper. The samples are centrifuged at 12 000 g for 10 minutes at 4°C. The supernatant, pull‐down complex or immunoprecipitated complex was mixed with 4 × SDS‐loading buffer (200 mmol/L Tris‐Cl (pH 6.8), 400 mmol/L DTT, 8% SDS, 4.0 mmol/L EDTA, 0.4% bromophenol blue, 40% glycerol) to denature. After boiling for 10 minutes, the samples are separated by SDS‐PAGE and transferred to PVDF membrane. The membrane was blocked with 5% skim milk or 3% bovine serum albumin^35^ in PBST (PSB with 0.1% Tween‐20) for 1 hour. The specific antibodies used included SIRT2 (Sigma), Hsp90 (Proteintech, IL), acetylated Hsp90 (Rockland Immunochemicals, PA), glucocorticoid receptor and acetylated protein (Abcam, MA), TNFα (Abcam), IL‐1 (Abcam), IL‐6 (Abcam), and β‐actin and lamin B1 (Proteintech). HRP‐conjugated antimouse and anti‐rabbit (Bio‐Rad) were used as secondary antibodies, and normal IgG (Santa Cruz) was used as a control. In brief, antibodies diluted in 5% skim milk or 3% BSA in PBST and incubated with PVDF membranes overnight at 4°C. The membranes were washed in PBST for 3 × 10 minutes with gentle shaking and then were incubated with secondary antibodies (1:2000 to 1:10 000) in 5% skim milk in PBST. After washing 3 times, protein bands were visualized using ECL immunoblot detection reagents, as previously described.^11^ All assays were repeated at least three separate times yielding similar results, and a representative result blots are shown.

### 2.11 Statistical analysis

Fluorescence value and qPCR data are presented as mean ± standard error of the mean (SEM). Statistical analysis was performed with Student's *t* test in GraphPad Prism. Significance was set at *P* < 0.05(*) or *P* < 0.01(**) as indicated.

## 3 RESULTS

### 3.1 Expression of GST‐SIRT2 in *E coli* with low solubility

**FIGURE 1:**
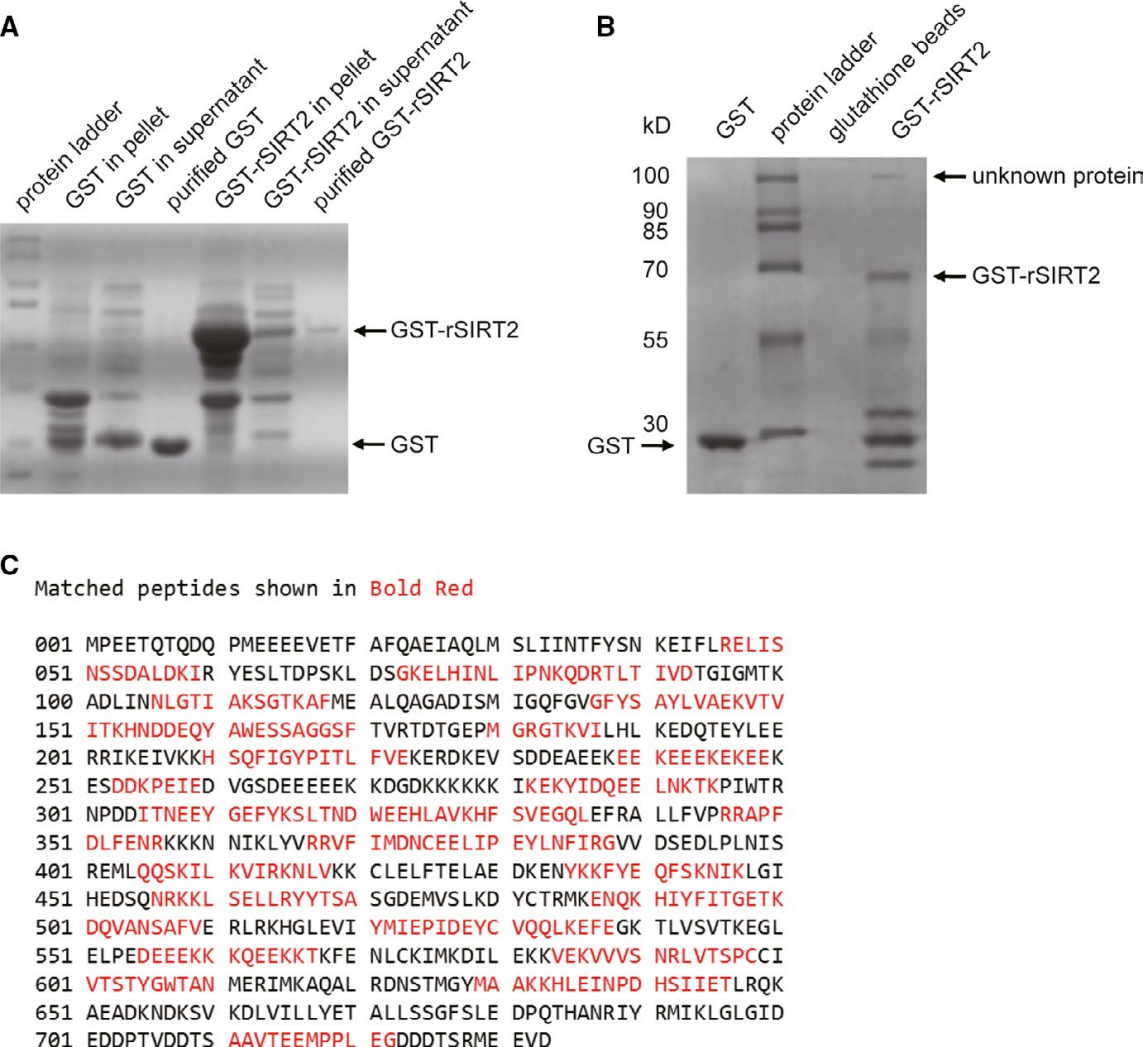
A, Rat SIRT2 (rSIRT2) coding region was cloned into pGEX‐6P‐1 expression vector. Analysis of expression and purification revealed that GST‐rSIRT2 predominantly expressed in inclusion bodies (pellet), but GST is mainly in soluble fraction (supernatant). Subsequently, both GST‐rSIRT2 and GST were purified from supernatant of the lysate. B, In a pull‐down assay, a new protein band was isolated with GST‐rSIRT2 compared with GST alone or glutathione beads. This unknown protein has an approximate molecular size of 100 kD. C, The new protein band was excised for identification by mass spectrometry, and results showed 48% sequence coverage with rat Hsp90α

GST‐SIRT2 and GST were expressed in *E coli*, and the solubility was assessed by SDS‐PAGE. Coomassie staining indicates that GST‐SIRT2 predominantly remained in inclusion bodies with only a small proportion was soluble in the supernatant (Figure 1A). In comparison, GST remains largely soluble. Only the soluble fraction of GST‐SIRT2 fusion protein was purified for use in the following experiments. In brief, both GST‐SIRT2 and GST are highly purified as shown in Figure 1A.

### 3.2 Hsp90α identified as a novel interacting partner of GST‐SIRT2

In the pull‐down assay, a new band was present in the GST‐SIRT2 lane in the Coomassie stained gel with a molecular weight around 100 kD (Figure 1B). There was no band visible at the corresponding molecular weight in either the glutathione bead or GST lanes, indicating a novel protein that interacts with SIRT2. Subsequently, this band was excised and sent for protein identification via mass spectrometry. A Mascot search of the NCBInr database identified this protein as rat Hsp90α (NP_786937) based on matched peptides corresponding to 48% sequence coverage (Figure 1C).

### 3.3 SIRT2 associates with Hsp90α in B104 cells

**FIGURE 2:**
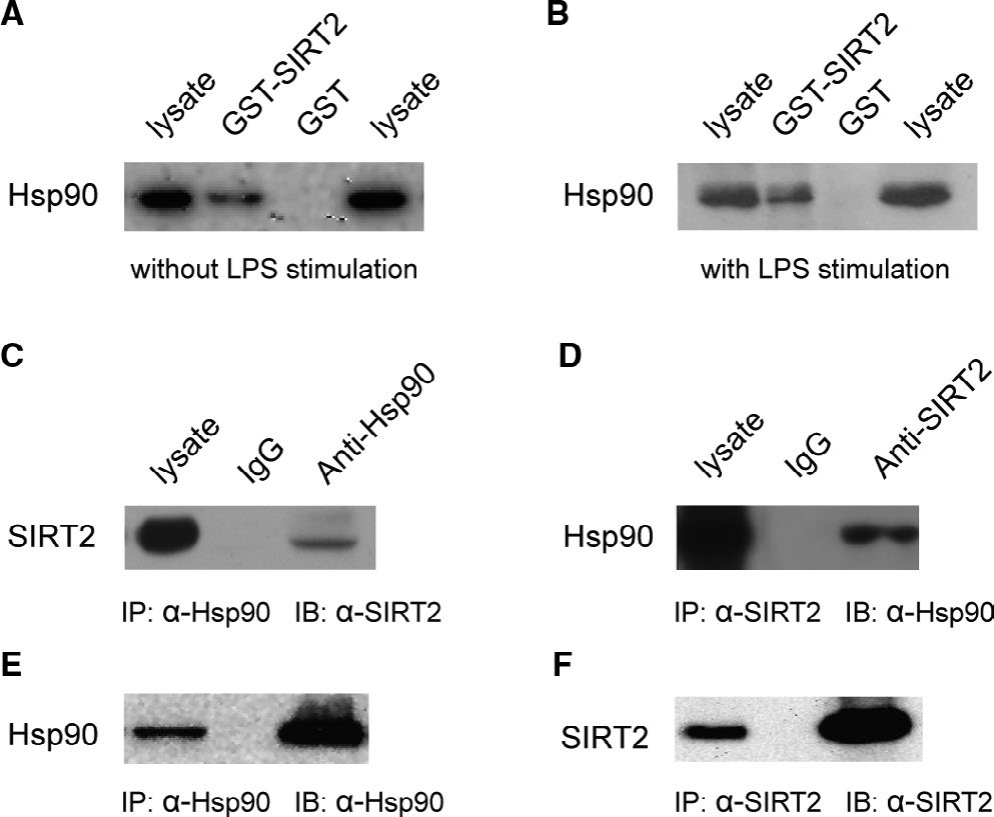
A pull‐down assay was performed with GST‐SIRT2 fusion protein from the lysate of B104 cells under control conditions (A) or treated with 10 ng/mL LPS for 12 h (B). Immunoblots showed Hsp90 interacts only with GST‐SIRT2, but not GST alone. LPS stimulation appears to promote binding of Hsp90 to SIRT2. Co‐immunoprecipitation was performed with anti‐Hsp90 (C and E) and anti‐Sirt2 (D and F). Immunoblots showed detection of SIRT2 in Hsp90 co‐IPs (C) and detection of Hsp90 in SIRT2 co‐IPs (D), suggesting that SIRT2 and Hsp90 form a complex in vivo. All assays were repeated at least three separate times yielding similar results, and representative immunoblots are shown. The precipitated protein complex was probed with anti‐Hsp90 (E) and anti‐SIRT2 (F) to confirm co‐IP with specific antibodies

To confirm a physical association between SIRT2 and Hsp90α, GST‐SIRT2 fusion protein was fixed on glutathione beads to pull‐down Hsp90α in B104 cells with or without LPS stimulation. GST‐SIRT2 pulled down Hsp90α from cell lysate in both stimulated and non‐stimulated cells compared with GST alone (Figure 2A,B); however, the association between SIRT2 and Hsp90α appears to increase in cells stimulated with LPS in the lane of GST‐SIRT2 (Figure 2B compared to A). Next, binding of endogenous SIRT2 to Hsp90α was examined by Co‐IP. As indicated in Figure 2C‐F, SIRT2 and Hsp90α can be detected in Co‐IP complexes. This was shown using anti‐Hsp90α for IP followed by detection of SIRT2 by immunoblot (Figure 2C), or using anti‐SIRT2 for IP followed by detection of Hsp90α by immunoblot (Figure 2D).

### 3.4 SIRT2 deacetylates Hsp90α in B104 cells

**FIGURE 3:**
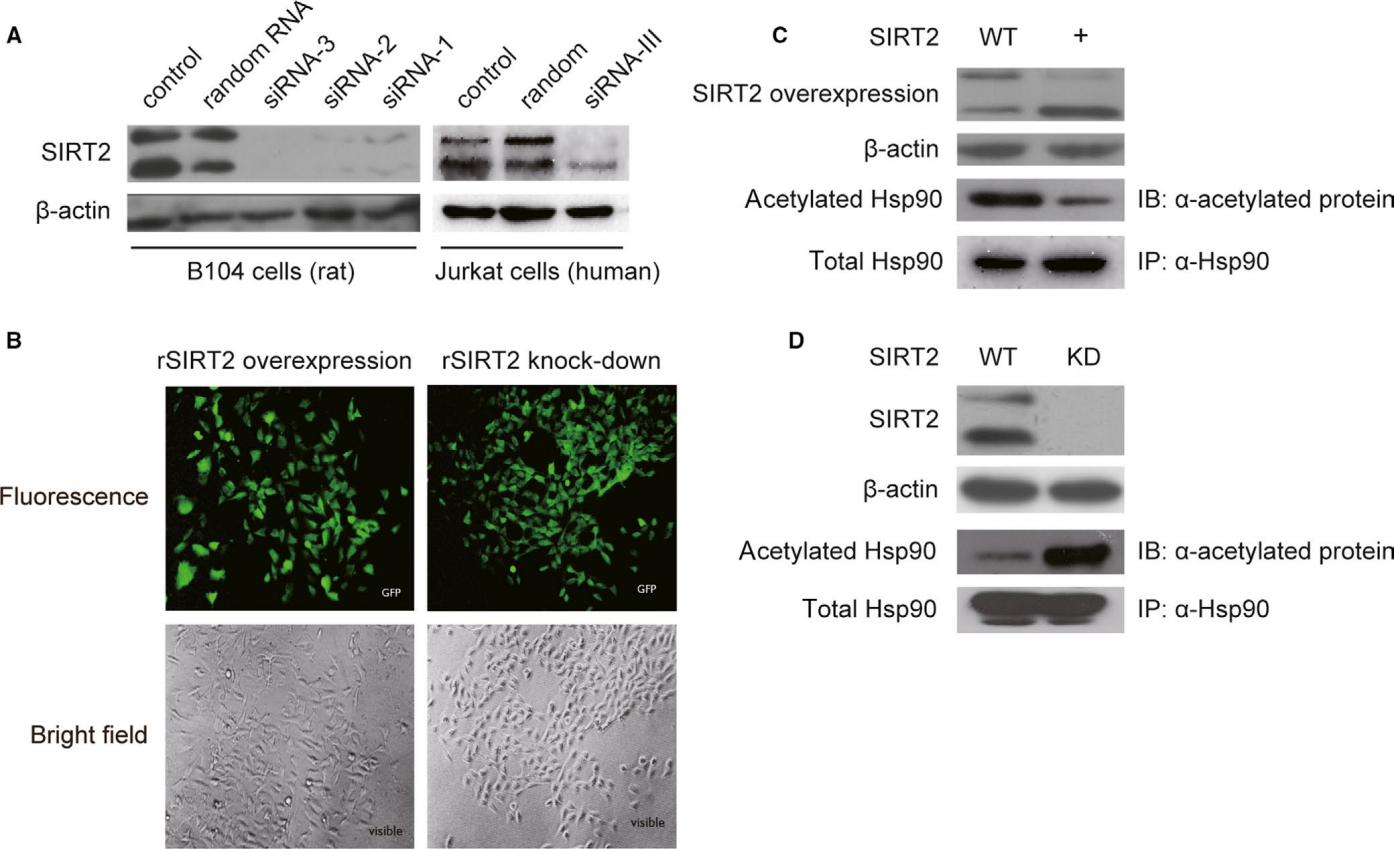
Three fragments of siRNA against rat SIRT2 (rSIRT2) mRNA were examined for efficiency of SIRT2 knock‐down. All fragments have an effective suppression in SIRT2 expression with siRNA‐3 being most effective (A; left panel). The corresponding sequence against human SIRT2 (hSIRT2) mRNA, derived from the siRNA‐3 and named siRNA‐III, was tested in Jurkat cells for SIRT2 knock‐down efficiency (A; right panel). Subsequently, both siRNA‐3 and siRNA‐III were used to construct lentiviral vectors. Viral particles for rSIRT2 overexpression (LV5 vector) and rSIRT2 knock‐down (pGLV3/H1/GFP + Puro vector) were infected into B104 cells with high efficiency (B). Co‐IP with anti‐Hsp90 in B104 cells overexpressing rSIRT2 (+) showed a decrease in the acetylation levels in the protein band corresponding to Hsp90 compared with control cells (WT) (C), whereas SIRT2 knock‐down^36^ showed an increase in the acetylation levels of the protein band corresponding to Hsp90 compared with control cells (WT) (D). Taken together, these results suggest that Hsp90 is a direct target of SIRT2 deacetylation. Hsp90 co‐IPs (C and D) were repeated three separate times, and a representative immunoblots are shown

To determine the functional relationship between SIRT2 and Hsp90α, B104 cells were infected by SIRT2 overexpression and knock‐down lentiviral constructs. For construction of lentiviral particles, three sequences of double‐stranded RNAs against rSIRT2 mRNA (siRNA 1‐3) were tested for efficiency. Western blot of lysates from transfected B104 cells showed that all three fragments down‐regulated SIRT2 expression with siRNA‐3 being the most effective (Figure 3A, left panel). Furthermore, transfection of siRNA‐III against the corresponding sequence of hSIRT2 results in the down‐regulation of SIRT2 expression in Jurkat cells (Figure 3A, right panel). Commercially constructed lentiviral particles were made with siRNA‐3 and siRNA‐III for knock‐down and SIRT2 cDNA for overexpression. rSIRT2 overexpression and knock‐down lentiviral vectors were infected into B104 cells, and detection of GFP positive cells indicates high infection efficiency (Figure 3B).

SIRT2 overexpression resulted in a decrease in acetylation level of Hsp90α (Figure 3C). Immunoprecipitation of B104 cell lysates with anti‐Hsp90α was performed to precipitate the total Hsp90 and examine levels of acetylated Hsp90α in infected (+) and control (WT) cells However, relative to total Hsp90α, the amount of acetylated Hsp90α was reduced in cells overexpressing SIRT2(Figure 3C). In contrast, knock‐down of SIRT2 expression increases acetylation level of Hsp90α (Figure 3D). Again, immunoprecipitation with anti‐Hsp90α showed similar expression levels of Hsp90α in infected^36^ and control (WT) B104 cells, but there is an increase in the amount of acetylated Hsp90α in cells where SIRT2 has been knocked‐down.

### 3.5 Deacetylation of Hsp90 via SIRT2 facilitates GR translocation to nuclei

**FIGURE 4:**
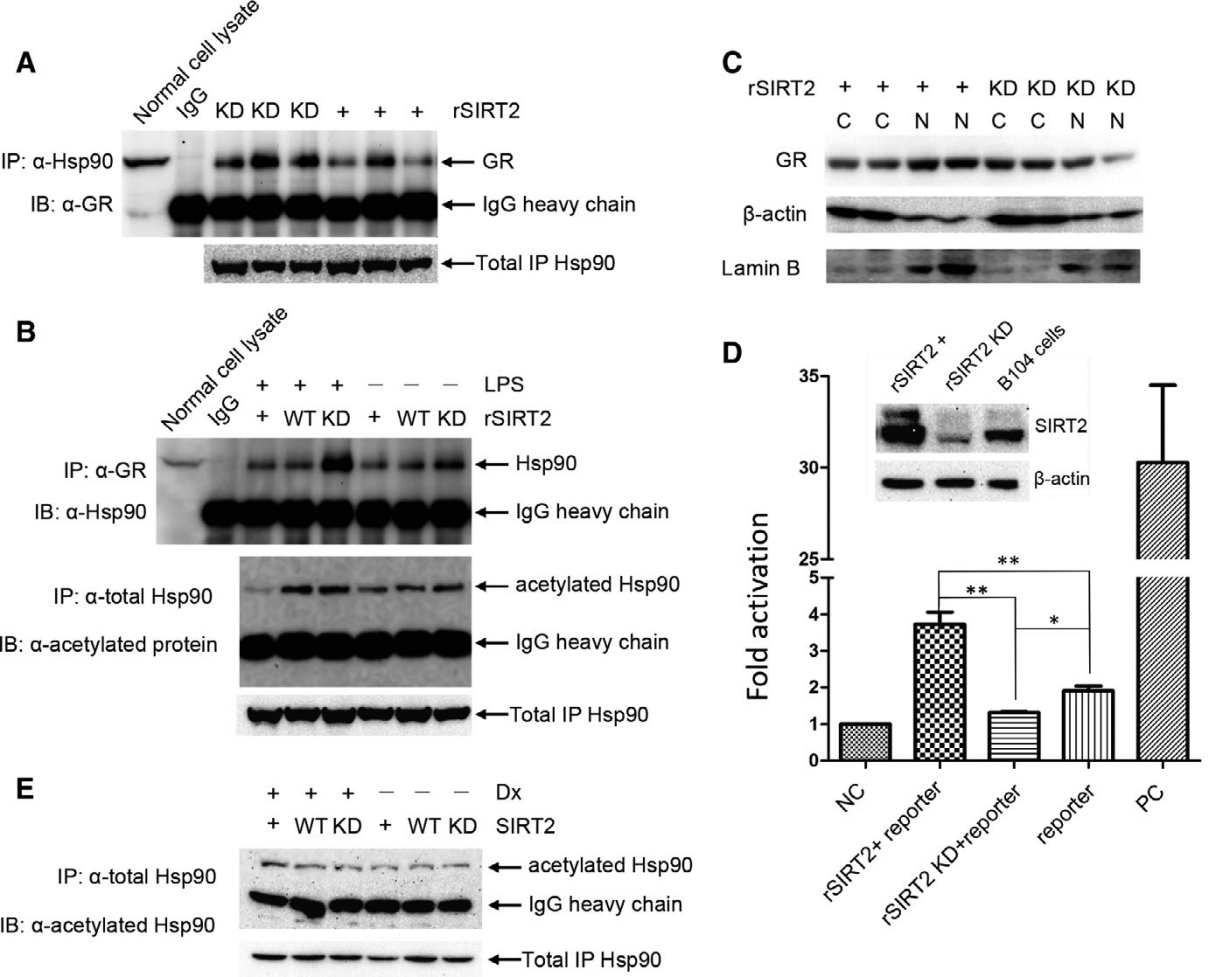
Co‐IP assays was carried out with anti‐Hsp90 in B104 cells in which SIRT2 was overexpression or knocked‐down, and glucocorticoid receptor (GR) was detected in the IP complex by immunoblot (A). SIRT2 overexpression resulted in decreased interaction between Hsp90 and GR compared with SIRT2 knock‐down. Co‐IP assays were also performed with anti‐GR in SIRT2 overexpression, SIRT2 knocked‐down or control cells, and Hsp90 was detected in the IP complex by immunoblot (B; top panel). Co‐IP with anti‐Hsp90 under the same conditions, followed by detection of acetylated Hsp90 (B; bottom panel), showed that the interaction between Hsp90 and GR corresponds to the acetylation levels of Hsp90. (C) GR subcellular location was detected in the SIRT2 overexpression or SIRT2 knocked‐down cells, with β‐actin and lamin B served as markers for the fractions of cytoplasm and nucleus, respectively. SIRT2 overexpression increased the relative expression of GR in the nucleus compared with SIRT2 knock‐down. Nuclei isolation was repeated at least three times, and a representative immunoblot is shown. (D) To examine the effect of SIRT2 on the binding capacity of GR to glucocorticoid receptor response element (GRE), GR‐driven dual‐luciferase reporter assay was performed in the B104 cells, in which rSIRT2 was overexpressed or knocked‐down,^50^ as described in the Materials and Methods. When rSIRT2 was overexpressed, expression of the reporter gene was significantly up‐regulated compared with rSIRT2 was knocked‐down or control cells (each experiment was performed in triplicate and represented as mean ± SEM; *t* test, *P < 0.05 or **P < 0.01). (E) The B104 cell or with SIRT2 overexpression or with SIRT2 knocked‐down were treated with 10 μmol/L of Dx for 12 h. The cell lysate was incubated with anti‐Hsp90 antibody to immunoprecipitate total Hsp90 protein. Subsequently, the acetylated Hsp90 was detected in the complex through Western blot

Previous reports have shown Hsp90, a molecular chaperone, interacts with the GR receptor.^37^ To investigate the role SIRT2 in regulating this interaction between Hsp90α and GR, IP assays were performed with anti‐Hsp90α in B104 cells with SIRT2 overexpression or knock‐down. SIRT2 overexpression, which leads to the deacetylation of Hsp90α, results in disassociation of Hsp90α and GR complex (Figure 4A, right three lanes labelled +) compared with SIRT2 knock‐down (Figure 4A, left three lanes labelled kD). The total cell lysate and normal IgG served as positive and negative controls, respectively. These data indicated that acetylation is required for Hsp90α association with its target protein. In cells stimulated with LPS, IP assays with anti‐GR antibody showed an increased association between Hsp90α and GR (Figure 4B, top panel). With or without LPS stimulation, SIRT2 knock‐down^36^ increased association between Hsp90α and GR compared with SIRT2 overexpression (+) or control cells (WT). SIRT2 knock‐down enhances binding of Hsp90α to GR compared with the same sample without LPS stimulation, but SIRT2 overexpression lowered the effect of LPS. Interestingly, the level of interaction between Hsp90α and GR corresponds to acetylation of Hsp90α (Figure 4B, bottom panel), presumably at K294 which has been previously identified as a critical acetylation site in the regulation of Hsp90α activity.^30^ SIRT2 overexpression decreased the acetylation level of Hsp90α compared with the control or SIRT2 knock‐down cells in IP assays performed with anti‐Hsp90α followed by immuno‐detection with an antibody for acetylated Hsp90.

Subsequently, the effect of SIRT2 on GR subcellular location was explored using nuclei and cytoplasm fractionation of cell lysates. SIRT2 overexpression facilitated GR translocation to nuclei, where GR was predominantly detected (Figure 4C, left lanes labelled +). In contrast, when SIRT2 expression was knocked‐down, GR was mainly localized in the cytoplasm (Figure 4C, right lanes labelled kD). Thus, SIRT2 expression altered the proportion of GR in nuclei versus cytoplasm. Taken together, these data indicate that SIRT2 deacetylates Hsp90 inducing its disassociation with GR, leading to GR translocation to nuclei. Nuclear translocation of GR would result in binding to the GRE (glucocorticoid response element) on genomic DNA and transcriptional activation or repression of downstream genes. To test effects of SIRT2 on the association of GR with GRE in B104 cells, a GR‐driven luciferase reporter assay was performed (Figure 4D). The results showed that SIRT2 overexpression enhanced reporter gene expression compared with control cells. Moreover, the fluorescence value is also greater than SIRT2 knocked‐down, suggesting that SIIRT2 effectively increase GR transcriptional activity. The negative and positive controls were transfected into naive B104 cells to confirm that the reporter system worked. Finally, dexamethasone (Dx) did not significantly reverse the increase of Hsp90 acetylation level induced by SIRT2 knock‐down. Conversely, the Dx reduced the difference of the acetylated Hsp90 between SIRT2 overexpression and knock‐down.

### 3.6 SIRT2 suppresses expression of inflammatory factors under LPS stimulation

**FIGURE 5:**
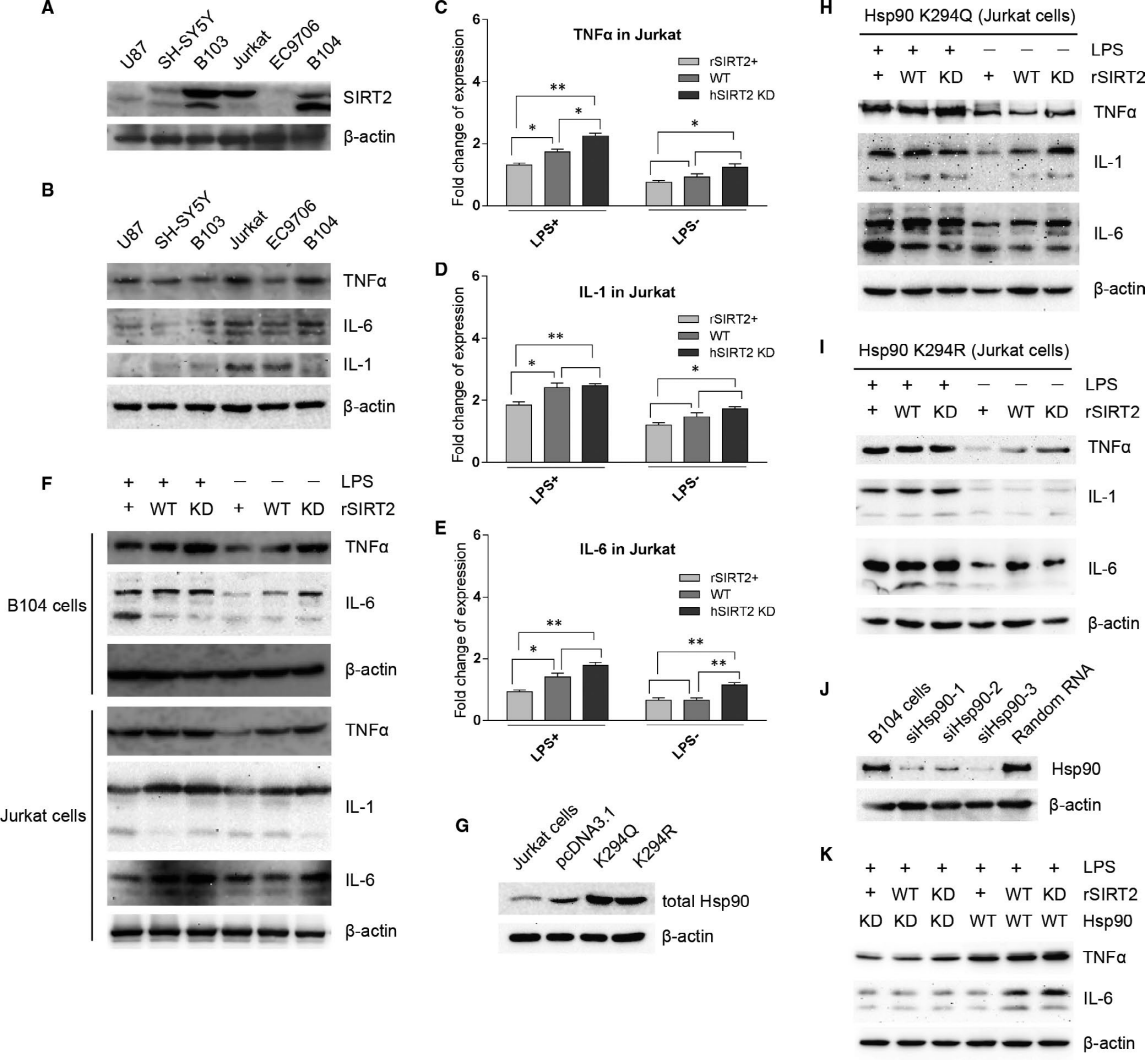
SIRT2 expression was examined in six cell lines. B103, B104 and Jurkat cells express SIRT2 (A). These cell lines were also tested for expression of TNFα, IL‐1 and IL‐6. The results showed that Jurkat cells express three cytokines and B104 cells express only TNFα and IL‐6 (B). qPCR for cytokine mRNA was performed in Jurkat cells (C‐E). rSIRT2 overexpression significantly reduced the mRNA levels of TNFα (C), IL‐1(D) and IL‐6(E) compared with hSIRT2 (siRNA‐III) knocked‐down, particularly for expression of cytokines under LPS stimulation (C‐E). In contrast, no significant change was observed between cells with hSIRT2 (siRNA‐III) knocked‐down and control cells for expression of IL‐1 under LPS stimulation (D), or between cells with rSIRT2 overexpression and control cells for expression of IL‐6 without LPS stimulation (E). qPCR was performed in triplicate plate for each sample, and data represent the mean ± SEM; *t *test, *P < 0.05 and **P < 0.01. Protein expression level of cytokines in Jurkat and B104 cells with SIRT2 overexpression, SIRT2 knocked‐down or control was detected with or without LPS stimulation (F). rHSP90 mutants K294Q and K294R were transfected into Jurkat cells, and overexpression compared with the control cells and blank vector was confirmed by Western blot (G). The acetylation‐mimic mutant K294Q reversed the repression of cytokine expression mediated by rSIRT2 overexpression (H), except for IL‐1 and IL‐6 without LPS stimulation. Conversely, acetylation‐null mutant K294R effectively reversed up‐regulation of cytokine expression mediated by rSIRT2 knock‐down (I), except for TNFα without LPS stimulation. To knock‐down Hsp90 expression in B104 cells, three RNA fragments were measured. siHsp90‐3 showed the most effective knock‐down of Hsp90 (J). Hsp90 knock‐down reduced cytokine expression levels regardless of SIRT2 expression under LPS stimulation (K)

Six cell lines were examined to further explore the relationship between SIRT2‐Hsp90‐GR signalling and inflammation. Western blot analysis demonstrated that B103, B104 and Jurkat cells expressed SIRT2 (Figure 5A). B104 cells express the inflammatory cytokines, TNFα and IL‐6, but not IL‐1. On the other hand, Jurkat cells express TNFα, IL‐6 and IL‐1 (Figure 5B). Therefore, these two cell lines were selected as cell models for LPS stimulation experiments. It is plausible to speculate that SIRT2 mainly affects the binding of GR to GRE around the promoters of the inflammatory cytokines, regulating transcription of the downstream genes. Hence, qPCR was performed to examine mRNA levels of cytokines in human Jurkat cells. The results demonstrated that SIRT2 overexpression significantly suppressed expression of the inflammatory cytokines compared with SIRT2 knocked‐down, particularly under LPS stimulation (Figure 5C,D,E). SIRT2 overexpression effectively decreased IL‐6 expression compared with SIRT2 knocked‐down even without LPS stimulation, but its effect is similar to WT (Figure 5E). Moreover, SIRT2 knocked‐down cells and control cells have similar expression levels of IL‐1 with LPS stimulation (Figure 5D). At the protein level, SIRT2 knock‐down facilitated expression of all three cytokines in Jurkat cells, as well as TNFα and IL‐6 in B104 (Figure 5F). LPS stimulation enhanced expression of these cytokines, but this phenomenon was not observed to the same degree with SIRT2 overexpression. SIRT2 overexpression significantly repressed expression of the inflammatory cytokines despite LPS stimulation (Figure 5C,D,E). In comparison, SIRT2 knock‐down resulted in a marked increase in expression of the inflammatory cytokines with or without LPS stimulation.

**FIGURE 6:**
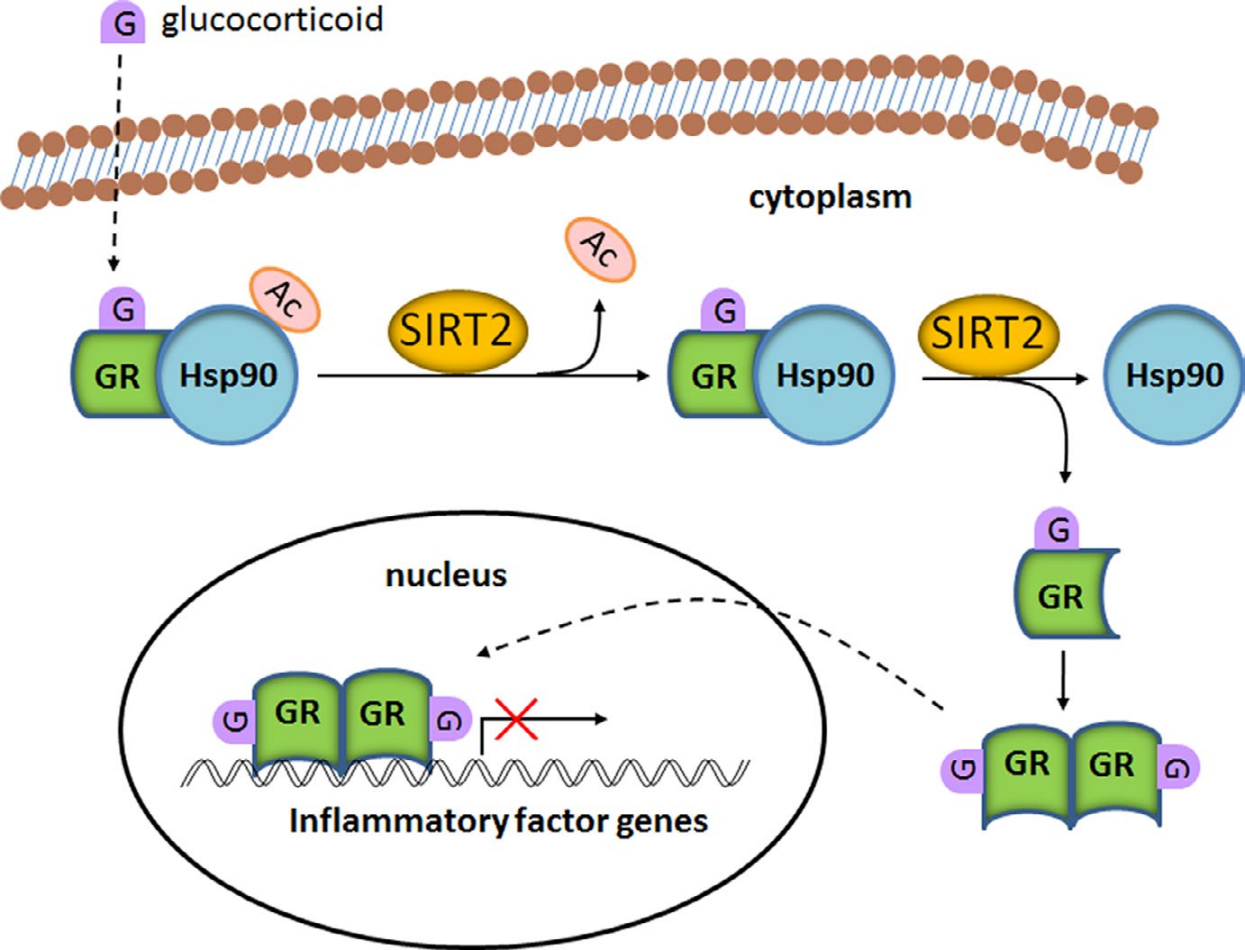
A schematic model that summarizes that SIRT2 regulation of Hsp90‐GR signalling. In resting state, Hsp90 and GR form a complex in the cytoplasm. The binding of glucocorticoid to GR triggers release of GR from the complex. SIRT2 facilitates this process by deacetylation Hsp90. Upon release, GR forms a homodimer and translocates into the nucleus, where GR preferentially binds to the GRE in the promoter region of inflammatory cytokines and represses the transcriptional activation

To determine whether the acetylation of Hsp90 K294 plays an important role in mediating expression of the inflammatory factors, rHsp90 K294 was mutated to either mimic constitutive acetylation (K294Q) or constitutive deacetylation (K294R). After transfection to Jurkat cells, we first confirmed both mutants were overexpressed (Figure 5G). Overall, the acetylation‐mimic mutation (K294Q) increased the expression levels of the inflammatory cytokines compared with the acetylation‐null mutation (K294R) both with and without LPS stimulation (Figure 5H‐I). We demonstrated that K294Q almost abolished the impact of SIRT2 overexpression and SIRT2 knocked‐down in Jurkat cells (Figure 5H) compared with wild‐type Hsp90 (Figure 5I). Similarly, K294R also abolished the effect of SIRT2 expression (Figure 5I). Interestingly, the effect of K294Q on cytokine expression is greater with LPS stimulation compared with control conditions (Figure 5H). This was also observed for K294R, but only for the expression of TNFα (Figure 5I). To examine significance of Hsp90 in SIRT2 mediating cytokine expression, Hsp90 expression was knocked‐down using siRNA (Figure 5J). We found that knock‐down of Hsp90 totally reduced expression of TNFα and IL‐6 in a SIRT2 independent manner (Figure 5K). These results support a repressor role for SIRT2 in the inflammatory response. As depicted schematically in Figure 6, we propose a novel signalling pathway by which SIRT2 regulates inflammation by repressing expression of the inflammatory cytokines through Hsp90‐GR signalling.

## 4 DISCUSSION

Protein acetylation and deacetylation of post‐translation modifications are important regulators of many cellular signalling pathways.^38^, ^39^ Deacetylases are classified into four classes based on sequence homology. Many of these deacetylases play a ubiquitously biological role in balancing the acetylation level of histone and non‐histone proteins involved in proliferation, differentiation, migration, metabolism and inflammation.^1^, ^21^, ^38^, ^40^ SIRT2, a member of the Sirtuin deacetylase family resembling Sir2 in yeast^41^ has recently been recognized as a regulator of inflammatory reaction.^19^, ^21^, ^42^

In the present study, Hsp90 has been identified as a substrate of SIRT2 through pull‐down and mass spectrometry, showing 48% sequence coverage to the rat Hsp90α (NP_786937). Hsp90 is a known molecular chaperone,^43^ and its interaction with the GR has well been characterized.^44^ Hsp90 plays a critical role in regulation of GR translocation to nuclei.^44^, ^45^ Therefore, we postulated that SIRT2‐dependent deacetylation of Hsp90 may have a critical effect on its ability to disassociate with GR. To substantiate this hypothesis, we demonstrate that SIRT2 regulated the level of Hsp90‐GR interaction in IP experiments using both anti‐Hsp90 and anti‐GR. Subsequently, SIRT2 overexpression, which led to a decrease in the association between Hsp90 and GR resulted in translocation of the GR to the nucleus. On the other hand, SIRT2 knock‐down, which led to an increase in the association between Hsp90 and GR resulted in GR remaining predominately in the cytoplasm. After Dx treated the cells with SIRT2 overexpression or knock‐down, we did not observe that Dx would significantly reduce the acetylation level of Hsp90 induced by SIRT2 deficiency, but the difference between SIRT2 overexpression and knock‐down was decreased. It is possible that glucocorticoid does not repress inflammatory factors expression through Hsp90 deacetylation. SIRT2 overexpression was found to increase binding of GR to GRE, and SIRT2 knocked‐down decreased binding of GR to GRE when compared with control cells. These results demonstrate that SIRT2 not only facilitated GR translocation into nucleus, but also modulates gene transcription via GR – GRE binding. A recent study suggested that SIRT2 deacetylated Hsp90 and facilitated Hsp90 degradation.^46^ In the present, we did not check Hsp90 protein level after SIRT2 overexpression of knock‐down, but we demonstrated that SIRT2 has an influence on the binding between Hsp90 and GR.

Interestingly, LSP stimulation led to an increase in the association between Hsp90 and GR. Furthermore, SIRT2 overexpression mitigated the effect of LPS down‐regulating the level of Hsp90‐GR interaction, while SIRT2 knock‐down facilitated Hsp90‐GR complex formation. Taken together, these data suggest that SIRT2 plays an important role in regulating association of Hsp90 and GR, particularly during the inflammatory response. The release of GR from the Hsp90 complex and subsequent translocation into the nucleus would regulate the expression of downstream inflammatory genes. Thus, we preferentially examined the impact of SIRT2 expression on the downstream expression of select cytokines in B104 cells, and then extended these studies to Jurkat human T lymphocyte cells, which was derived from human T cell leukaemia and is widely used for analysis of cytokine production.^47^ LPS stimulation up‐regulated the expression of TNFα, IL‐1 and IL‐6 mRNA in Jurkat cells. While SIRT2 overexpression decreased levels of the inflammatory cytokines in both stimulated and non‐stimulated cells, this appears to be more prominent under inflammatory conditions. Conversely, SIRT2 knock‐down enhanced expression of these cytokines. Thus, a similar pattern of SIRT2 regulation was observed both in mRNA and protein expression levels of the inflammatory cytokine. A summary of the results in Figure 6 depicts the molecular mechanism by which SIRT2 can regulate the inflammatory response through SIRT2‐Hsp90‐GR‐cytokine repression.

Recently, Min et al reported that SIRT2 deacetylated Hsp90 and facilitated Hsp90 degradation.^46^ Deacetylation of Hsp90 at K294 was previously reported to alter its binding to GR,^30^ which is likely to be a critical site for deacetylation via SIRT2. The acetylation‐mimic mutant almost abolished the repression of cytokine expression mediated by SIRT2 overexpression under LPS stimulation. In contrast, in non‐stimulated cells, this mutant had a minimal impact on SIRT2 repression. The acetylation‐null mutant abolished the impact of SIRT2 on cytokine expression in both stimulated and non‐stimulated cells. Therefore, these results indicated that Hsp90 K294 is an important acetylation site involving in regulation expression of the inflammatory cytokines. Interestingly, cytokine expression is still detectable with overexpression of the dominant‐negative K294R mutant, although to a lesser degree than with overexpression of K294Q. This may be due to expression of endogenous Hsp90, or may by mediated via other signalling pathway(s). This is indicative of a robust modulatory role for SIRT2‐dependent Hsp90 deacetylation in the response to inflammatory stimuli, but a lesser influence of this pathway under normal conditions. Finally, as mediator, Hsp90 plays a critical role in SIRT2‐Hsp90‐GR axis. Hsp90 knock‐down reduced TNFα and IL‐6 expression regardless of SIRT2 overexpression or knock‐down.

Several other studies also indicate that SIRT2 deacetylase activity modulates the inflammatory reaction.^21^, ^22^ For example, SIRT2 inhibits inflammation in animal models of collagen‐induced arthritis, sepsis, or colitis.^16^, ^22^, ^23^, ^24^ More recently, SIRT2 mRNA expression levels of extracellular plasma was found to be reduced in 54 rheumatoid arthritis patients compared with healthy controls.^17^ It is possible that a resultant decrease in SIRT2 protein expression contributed to the up‐regulated expression and release of inflammatory factors. As a result, inflammation was exacerbated. NF‐kB has been identified as a target substrate of SIRT2 in modulating inflammation^22^, ^23^, ^24^; however, in many studies, the precise molecular mechanism of SIRT2 action remains unknown. Here, we have characterized a novel pathway by which SIRT2 influences the inflammatory response (Figure 6). Based on our finding, we postulate that deacetylation of Hsp90 releases GR allowing it to dimerize and translocate to the nucleus. GR binding to GRE, or possibly by also forming a heterocomplex with NF‐kB,^26^ inhibits the transcriptional activation of inflammatory cytokines.

A mild inflammatory reaction is necessary for an organism to fight infections and remove pathogens, but excessive inflammation may be detrimental. Sirtuins may play a critical role in balancing the extent of an inflammatory reaction. A growing body of evidence suggests that SIRT2 down‐regulates excessive inflammation via multiple and partially redundant mechanisms. Intriguingly, it has been reported that loss of SIRT1 led to down‐regulation of inflammatory cytokine gene expression^48^ while treatment with a SIRT1 agonist was able to overcome immunosuppression^49^ suggesting that SIRT1 enhances the inflammatory reaction. Thus, SIRT1 and SIRT2 may have apposing effects on inflammation, adding another level of complexity to the control Sirtuin family of deacetylases exerting over cellular function. In the future, SIRT2 may have potential as a treatment target to regulate the exacerbated immune responses associated with inflammatory‐related diseases.

## CONFLICT OF INTEREST

All authors declare that they have no conflict of interests with the contents of this article.

## AUTHOR CONTRIBUTIONS

K. Sun and X. Wang performed almost all the experiments; N. Fang, W. Zhao and Y. Lin took part in gene cloning, real‐time PCR and GRE‐driven dual‐luciferase assays; A. Xu and X. Zhao performed protein expression and purification for pull‐down and participated in construction of the recombinant vectors, cell culture and protein assays; Aj. Nazarali contributed to design assays and discussed the results. S. Ji designed the project and interpreted the data.

## ETHICAL APPROVAL

This research article does not contain any investigations or studies with human participants or animals performed by any author.

## Data Availability

The data that support the findings of this study are available from the corresponding author upon reasonable request.
